# Supplementary material for: Quantitative Analysis of Acetone in Transformer Oil Based on ZnO NPs@Ag NWs SERS Substrates Combined with a Stoichiometric Model
Source: Int J Mol Sci. 2022 Nov 7;23(21):13633. doi: 10.3390/ijms232113633 (PMC9658008; doi:10.3390/ijms232113633)
Supplement: Supplementary file 1 [file ijms-23-13633-s001.zip › ijms-1927589-SI.pdf]

Supporting Information

Quantitative analysis of acetone in transformer oil based on ZnO NPs@Ag NWs SERS substrates combined with stoichiometric model

Xinyuan Zhang , Yu Lei, Ruimin Song, Weigen Chen*, Changding Wang, Ziyi Wang, Zhixian Yin and Fu Wan

State Key Laboratory of Power Transmission Equipment & System Security and New Technology, Chongqing University, Chongqing 400044, China

* Correspondence: weigench@cqu.edu.cn (W.C.)

S1. TEM of pristine Ag NWs

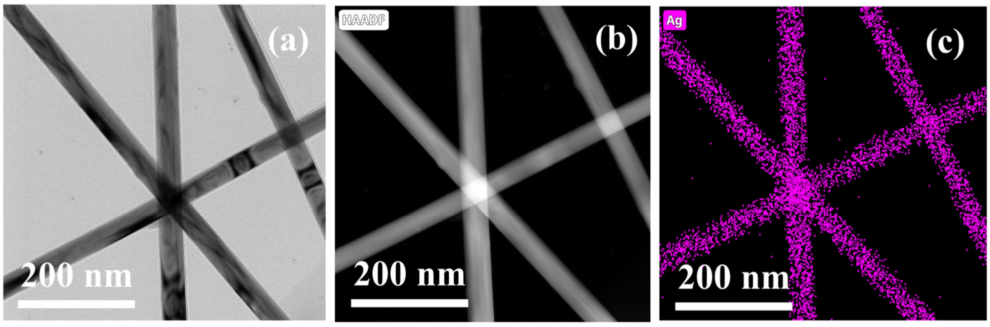

Figure S1. TEM of pristine Ag NWs. TEM image (**a**), HAADF (**b**), and EDS mapping images (**c**) of pristine Ag NWs.

S2. Calculation of EF value

We used R6G as a probe molecular (probe molecular) to evaluate the enhancement effect of AgNWs@ZnO. The enhancement factor (EF) was calculated by the following equation:

$$EF_{SERS} = \frac{I_{SERS} / N_{SERS}}{I_{out} / N_{out}} \tag{4}$$

where I_{SERS} and I_{out} are the effective intensities of the characteristic peaks with and without ZnO NPs@Ag NWs substrates for the probe molecules, respectively, N_{SERS} and N_{out} are the numbers of the probe molecules within detection volume on the substrates with and without ZnO NPs@Ag NWs, respectively.

As in Figure S2, with 1650 cm^{-1} as the Raman characteristic peak, the intensity I_{SERS} for 10^{-6} M R6G on the SERS substrates is about 528.498 with ZnO NPs@Ag NWs, and the intensity I_{out} for 10^{-6} M R6G on the Si substrate is about 12.5582 without ZnO NPs@Ag NWs.

Since the Raman measurement and the conditions of the preparation are the same for substrates with and without ZnO NPs@Ag NWs, thus they have identical detection volumes and similar surface areas for molecules absorbed. Therefore, N_{SERS} and N_{out} are mainly determined by the concentration of R6G. The calculated value of $N_{SERS} / N_{out} = C_{SERS} / C_{out} = 10^6$.

Thus, the *EF* @1649cm^{-1} of the ZnO NPs@Ag NWs substrates is estimated to be 4.21×10^7.

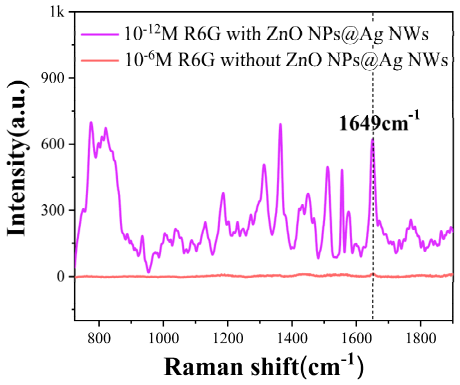

Figure S2. SERS spectrum of 10^{-12} M R6G with ZnO NPs@Ag NWs substate and Raman spectrum of 10^{-6} M R6G without ZnO NPs@Ag NWs

S3. Schematic diagram for the CT process

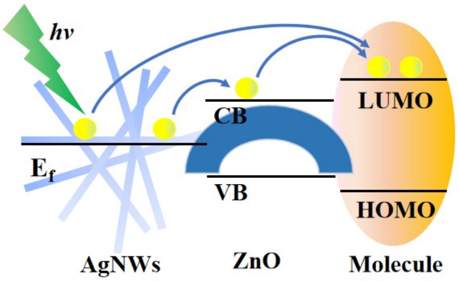

Figure S3. Schematic diagram for the CT process between ZnO, AgNWs, and the probe molecules

S4. SERS mapping

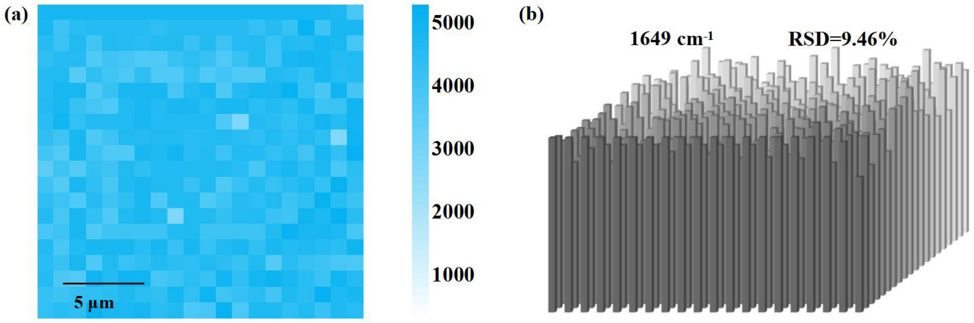

Figure S4. (**a**) SERS map of R6G at 1649 cm^{-1}; and corresponding histograms (**b**) for the above mapping area.

Raman mapping spectra was obtained by using a Renishaw in Via Raman microscope with a 600 lines/mm grating and a 514 nm laser. The incident laser beam was focused by a 50 ×objective and the laser power on the samples was kept 0.5 mW to avoid laser induced heating.

S5. Original SERS spectra

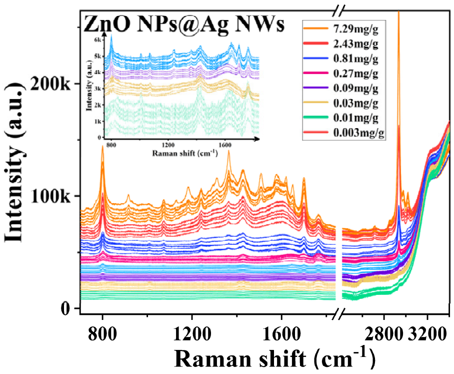

Figure S5. Original SERS spectra of different concentration acetone extracts (The inset shows the local magnification of 0.09 mg/g, 0.03 mg/g, 0.01 mg/g and 0.003 mg/g).

S6. Evaluation parameters of PLS models with different spectral preprocessing methods.

Table S1. Evaluation parameters of PLS models with different spectral preprocessing methods.

preprocessing method	PCs	R_c	RMSECV	R_p	RMSEP
None	4	0.214842	0.992273	0.980813	0.327962
SG	4	0.213069	0.992400	0.982357	0.314489
D1	3	0.348660	0.979649	0.975247	0.372506
SNV	3	0.175760	0.994828	0.992777	0.201215
MSC	3	0.219932	0.991902	0.987239	0.267463
Baseline	5	0.201398	0.993209	0.991935	0.212600
SG + D1	3	0.312821	0.983617	0.9777	0.35356
SG + SNV	4	0.163031	0.995550	0.994466	0.176127
SG + MSC	3	0.199619	0.993329	0.989236	0.245646
Baseline + SG	5	0.191552	0.993857	0.98547	0.285396
Baseline + D1	3	0.308613	0.984055	0.977152	0.357880
Baseline + SNV	5	0.128131	0.997251	0.997678	0.114081
Baseline + MSC	3	0.217877	0.992053	0.996586	0.138331
